# Supplementary material for: Microtomographic investigation of a large corpus of cichlids
Source: PLoS One. 2023 Sep 27;18(9):e0291003. doi: 10.1371/journal.pone.0291003 (PMC10529598; doi:10.1371/journal.pone.0291003)
Supplement: S1 File — The three-dimensional view of sample 104016 was generated in the otolith extraction notebook and saved as a self-contained HTML file with K3D-jupyter. A copy of this HTML file can be viewed and interacted with through the GitHub HTML preview. (HTML) [file pone.0291003.s002.html]

K3D snapshot viewer - [TIMESTAMP]
