# Supplementary material for: Microtomographic investigation of a large corpus of cichlids
Source: PLoS One. 2023 Sep 27;18(9):e0291003. doi: 10.1371/journal.pone.0291003 (PMC10529598; doi:10.1371/journal.pone.0291003)
Supplement: Attachment — Submitted filename: micro.pdf [file pone.0291003.s003.pdf]

PLOS ONE

Microtomographic investigation of a large corpus of cichlids

--Manuscript Draft--

**Manuscript Number:**	PONE-D-23-10896
**Article Type:**	Research Article
**Full Title:**	Microtomographic investigation of a large corpus of cichlids
**Short Title:**	Microtomographic investigation of a large corpus of cichlids
**Corresponding Author:**	David Haberthür, Ph.D<br>Universität Bern: Universitat Bern<br>Bern, SWITZERLAND
**Keywords:**	imaging<br>fish<br>ecology<br>tomography<br>image processing<br>python
**Abstract:**	A large collection of cichlids from Lake Victoria in Africa spanning a length range of 6 to 18 cm was nondestructively imaged using micro-computed tomography.<br>We describe our method to efficiently obtain three-dimensional tomographic datasets of the oral and pharyngeal jaws and the whole skull of these fishes for accurately describing their morphology.<br>The tomographic data we acquired (9.5 TB of projection images) yielded 1.4 TB of three-dimensional image stacks used for extracting the relevant features of interest.<br>Herein we present our method and an outlook on analyzing the acquired data; a morphological description of the oral and pharyngeal jaws of the fishes, a three-dimensional geometric morphometrics analysis of landmark features on the fish skulls, and a robust method to automatically extract the otoliths of the fishes from the tomographic data.
**Order of Authors:**	David Haberthür, Ph.D
	Mikki Law
	Kassandra Ford
	Marcel Häsler
	Ole Seehausen
	Ruslan Hlushchuk
**Additional Information:**	
**Question**	**Response**
**Financial Disclosure**<br><br>Enter a financial disclosure statement that describes the sources of funding for the work included in this submission. Review the submission guidelines for detailed requirements. View published research articles from *PLOS ONE* for specific examples.<br><br>This statement is required for submission and **will appear in the published article** if the submission is accepted. Please make	The authors received no specific funding for this work.

Powered by Editorial Manager® and ProduXion Manager® from Aries Systems Corporation

sure it is accurate.<br><br>**Unfunded studies**<br>Enter: *The author(s) received no specific funding for this work.*<br><br>**Funded studies**<br>Enter a statement with the following details:<br>• Initials of the authors who received each award<br>• Grant numbers awarded to each author<br>• The full name of each funder<br>• URL of each funder website<br>• Did the sponsors or funders play any role in the study design, data collection and analysis, decision to publish, or preparation of the manuscript?<br>• **NO** - Include this sentence at the end of your statement: *The funders had no role in study design, data collection and analysis, decision to publish, or preparation of the manuscript.*<br>• **YES** - Specify the role(s) played.<br><br>* typeset	
**Competing Interests**<br><br>Use the instructions below to enter a competing interest statement for this submission. On behalf of all authors, disclose any competing interests that could be perceived to bias this work—acknowledging all financial support and any other relevant financial or non-financial competing interests.<br><br>This statement is required for submission and **will appear in the published article** if the submission is accepted. Please make sure it is accurate and that any funding sources listed in your Funding Information later in the submission form are also declared in your Financial Disclosure statement.<br><br>View published research articles from *PLOS ONE* for specific examples.	The authors have declared that no competing interests exist.

**NO authors have competing interests**<br><br>Enter: *The authors have declared that no competing interests exist.*<br><br>**Authors with competing interests**<br><br>Enter competing interest details beginning with this statement:<br><br>*I have read the journal's policy and the authors of this manuscript have the following competing interests: [insert competing interests here]*<br><br>* typeset	
**Ethics Statement**<br><br>Enter an ethics statement for this submission. This statement is required if the study involved:<br><br>• Human participants<br>• Human specimens or tissue<br>• Vertebrate animals or cephalopods<br>• Vertebrate embryos or tissues<br>• Field research<br><br>Write "N/A" if the submission does not require an ethics statement.<br><br>General guidance is provided below. Consult the submission guidelines for detailed instructions. **Make sure that all information entered here is included in the Methods section of the manuscript.**	The study used specimens from an already existing fish collection, built over decades of research led by Ole Seehausen and continuing until today.<br>All relevant permits for research, the permits for export from the respective country and import to Switzerland were given and the research has followed modern ethical guidelines for scientific research.

Powered by Editorial Manager® and ProduXion Manager® from Aries Systems Corporation

**Format for specific study types**<br><br>**Human Subject Research (involving human participants and/or tissue)**<br>• Give the name of the institutional review board or ethics committee that approved the study<br>• Include the approval number and/or a statement indicating approval of this research<br>• Indicate the form of consent obtained (written/oral) or the reason that consent was not obtained (e.g. the data were analyzed anonymously)<br><br>**Animal Research (involving vertebrate animals, embryos or tissues)**<br>• Provide the name of the Institutional Animal Care and Use Committee (IACUC) or other relevant ethics board that reviewed the study protocol, and indicate whether they approved this research or granted a formal waiver of ethical approval<br>• Include an approval number if one was obtained<br>• If the study involved *non-human primates*, add *additional details* about animal welfare and steps taken to ameliorate suffering<br>• If anesthesia, euthanasia, or any kind of animal sacrifice is part of the study, include briefly which substances and/or methods were applied<br><br>**Field Research**<br><br>Include the following details if this study involves the collection of plant, animal, or other materials from a natural setting:<br>• Field permit number<br>• Name of the institution or relevant body that granted permission	
**Data Availability**<br><br>Authors are required to make all data underlying the findings described fully available, without restriction, and from the time of publication. PLOS allows rare exceptions to address legal and ethical concerns. See the PLOS Data Policy and FAQ for detailed information.	Yes - all data are fully available without restriction

A Data Availability Statement describing where the data can be found is required at submission. Your answers to this question constitute the Data Availability Statement and **will be published in the article**, if accepted.<br><br>**Important:** Stating 'data available on request from the author' is not sufficient. If your data are only available upon request, select 'No' for the first question and explain your exceptional situation in the text box.<br><br>Do the authors confirm that all data underlying the findings described in their manuscript are fully available without restriction?	
**Describe where the data may be found in full sentences. If you are copying our sample text, replace any instances of XXX with the appropriate details.**<br><br>• If the data are **held or will be held in a public repository**, include URLs, accession numbers or DOIs. If this information will only be available after acceptance, indicate this by ticking the box below. For example: *All XXX files are available from the XXX database (accession number(s) XXX, XXX.)*.<br>• If the data are all contained **within the manuscript and/or Supporting Information files**, enter the following: *All relevant data are within the manuscript and its Supporting Information files.*<br>• If neither of these applies but you are able to provide **details of access elsewhere**, with or without limitations, please do so. For example:<br><br>*Data cannot be shared publicly because of [XXX]. Data are available from the XXX Institutional Data Access / Ethics Committee (contact via XXX) for researchers who meet the criteria for access to confidential data.*<br><br>*The data underlying the results presented in the study are available from (include the name of the third party*	All relevant data are within the manuscript and its Supporting Information files.

and contact information or URL).<br>• This text is appropriate if the data are owned by a third party and authors do not have permission to share the data.<br><br>* typeset	
Additional data availability information:	

Powered by Editorial Manager® and ProduXion Manager® from Aries Systems Corporation

Manuscript file including the images

Click here to access/download;Manuscript;manuscript.e1e2ef7.pdf

Microtomographic investigation of a large corpus of cichlids

A preprint of this manuscript is available at bioRχiv with the doi:10.1101/2023.03.30.534917. We submitted `v1.0` of the manuscript to Review Commons, aiming for PLOS ONE. The version you see here has been updated since submission.

This manuscript (permalink) was automatically generated from habi/EAWAG-manuscript@e1e2ef7 on April 24, 2023.

Authors

- **David Haberthür**
  0000-0003-3388-9187 · habi · habi
  Institute of Anatomy, University of Bern, Baltzerstrasse 2, CH-3012 Bern, Switzerland

- **Mikki Law**
  mlaw-migalig
  Aquatic Ecology and Evolution, Institute of Ecology and Evolution, University of Bern, CH-3012 Bern, Switzerland; Department of Fish Ecology and Evolution, Eawag, Swiss Federal Institute for Aquatic Science and Technology, CH-6047 Kastanienbaum, Switzerland

- **Kassandra Ford**
  0000-0002-4413-2633 · kassthefish
  Department of Biological Sciences, George Washington University, USA; Department of Fish Ecology and Evolution, Eawag, Swiss Federal Institute for Aquatic Science and Technology, CH-6047 Kastanienbaum, Switzerland; Aquatic Ecology and Evolution, Institute of Ecology and Evolution, University of Bern, CH-3012 Bern, Switzerland

- **Marcel Häsler**
  mphaesler
  Aquatic Ecology and Evolution, Institute of Ecology and Evolution, University of Bern, CH-3012 Bern, Switzerland; Department of Fish Ecology and Evolution, Eawag, Swiss Federal Institute for Aquatic Science and Technology, CH-6047 Kastanienbaum, Switzerland

- **Ole Seehausen**
  0000-0001-6598-1434 · OleSeehausen
  Aquatic Ecology and Evolution, Institute of Ecology and Evolution, University of Bern, CH-3012 Bern, Switzerland; Department of Fish Ecology and Evolution, Eawag, Swiss Federal Institute for Aquatic Science and Technology, CH-6047 Kastanienbaum, Switzerland

- **Ruslan Hlushchuk**
  0000-0002-6722-8996 · RuslanHlushchuk
  Institute of Anatomy, University of Bern, Baltzerstrasse 2, CH-3012 Bern, Switzerland

Abstract

A large collection of cichlids from Lake Victoria in Africa spanning a length range of 6 to 18 cm was nondestructively imaged using micro-computed tomography. We describe our method to efficiently obtain three-dimensional tomographic datasets of the oral and pharyngeal jaws and the whole skull of these fishes for accurately describing their morphology. The tomographic data we acquired (9.5 TB of projection images) yielded 1.4 TB of three-dimensional image stacks used for extracting the relevant features of interest. Herein we present our method and an outlook on analyzing the acquired data; a morphological description of the oral and pharyngeal jaws of the fishes, a three-dimensional geometric morphometrics analysis of landmark features on the fish skulls, and a robust method to automatically extract the otoliths of the fishes from the tomographic data.

Introduction

History

Cichlid fish in African lakes are powerful model systems in speciation and adaptive evolutionary radiation research [1,2]. The functional decoupling of their oral and pharyngeal jaws is hypothesized to be a factor in making cichlids unusually versatile in their feeding and allowing them the ability to adapt to a wide range of environmental factors [3]. The hypothesis is that the fusion of the lower pharyngeal jaws makes that jaw system a powerful food processing tool, in turn releasing the oral jaws from functional constraint. The oral jaws no longer need to process prey and can therefore specialize on prey capture.

Even though the evolutionary diversification of cichlid fish radiations in Lake Victoria in Africa is a well-researched issue it remains a complex system in need of further study [2,4]. We aim to better understand the functional anatomy of the skulls and jaws in these fish in order to test the functional decoupling and other hypotheses about what may facilitate exceptionally high rates of morphological evolution.

The collection of cichlid fish available is extremely valuable, hence a nondestructive imaging method is paramount for studying these samples. Since micro–computed tomography can be regarded as nondestructive method for biological samples, it is the ideal imaging method for investigating the oral and pharyngeal jaws as well as the skull features of the fish presented in this study [5].

Micro-computed tomography

X-ray microtomography is a valuable tool to gain insights into the inner structure of highly diverse samples, namely for specimens related to research done in the biomedical sciences. Microtomographic imaging has been employed as a method of choice to nondestructively assess the morphology of different fish species, large and small [5]. For a small overview of analyses which are possible with X-ray microtomographic imaging in relation to fish biology and morphology, see prior work of the authors of this manuscript [5–7] as well as other studies [8].

Depending on the structures of interest, biomedical samples are often only tomographically scanned after being stained with a contrast agent, most often employing contrast agents containing heavy metals. Since the structures of interest for the two studies we touch upon in this manuscript (cichlid teeth and skulls) display large enough contrast to the surrounding tissue we did not stain our samples prior to the tomographic imaging presented here.

Materials, Methods and Results

Sample procurement and preparation

The fish were kept in 75% ethanol for long-term storage at the Swiss Federal Institute of Aquatic Science and Technology (Eawag). They were delivered to the Institute of Anatomy for micro-CT imaging sorted into batches of approximately equal length.

Ethics statement

The study used specimens from an already existing fish collection, built over decades of research led by Ole Seehausen and continuing until today. All relevant permits for research, the permits for export from the respective country and import to Switzerland were given and the research has followed modern ethical guidelines for scientific research.

Micro-computed tomographic imaging

All samples were scanned on two of the three available high-resolution micro-CT machines of the Institute of Anatomy of the University of Bern in Switzerland, a SkyScan 1272 and a SkyScan 2214 (both Bruker microCT, Kontich, Belgium).

The fish were sorted into 'bins' based on their physical size. We used a custom-made sample-holder to scan each of the fish in our machines. It was 3D-printed on a Form 2 desktop stereolithography printer (Formlabs, Somerville, Massachusetts, USA), the file for printing the holder is available online [9] as part of a library of sample holders for tomographic scanning of biomedical samples [10]. The original *OpenSCAD* [11] file [12] is parametrized to effortlessly generate a file for 3D-printing sample holders to accommodate the varying width, height and length classes of the fish.

In total, we acquired 362 tomographic scans of 129 different specimens. All the scanning parameters are collected in a table in the Supplementary Materials; a generalized rundown is given below.

Since the fish greatly varied in their length (specimen length varied between 6 cm and 18 cm), the voxel sizes of each of the acquired datasets also varies greatly. We acquired datasets with (isometric) voxel sizes ranging from 3.5 to 50 μm.

Depending on the size of the specimen we set the X-ray source voltage to 50–80 kV and—depending on the voltage—to a current between 107 and 200 μA. The X-ray spectrum was filtered either by an aluminum filter of varying thickness (either 0.25, 0.5 or 1 mm for increasing specimen size) before digitization to projection images or recorded in an unfiltered way (for smaller specimen). In total we recorded 9.5 TB of projection images (`TIFF` and `*.iif` files, where the `*.iif` files are for the so-called alignment scans).

All the recorded projection images were subsequently reconstructed into virtual 3D stacks of axial `PNG` images spanning the regions of interest of each fish. All the specimens were scanned with their mouths facing downward in the sample holder and rotating along their long axis. We thus manually aligned each of the reconstructed datasets so that the lateral axis of the fish was horizontal in relation to the x and y direction of the reconstructed slices. We reconstructed the projection images with NRecon (Version 1.7.4.6, Bruker microCT, Kontich, Belgium) with varying ring artifact and beam hardening correction values, depending on each fish (again, all relevant values are listed in the Supplementary Materials). In total, this resulted in 1.4 TB of reconstruction images (nearly one million

`*rec*.png` files). Each of the 362 scans we performed has on average about 2700 reconstruction images.

While performing the work, a subset of the data was always present on the production system, for working with it (see Preparation for analysis below). A small bash script [13] was used to generate redundant (archival) copies of the raw projection images and copy all the files to a shared network drive on the 'Research Storage' infrastructure of the University of Bern, enabling collaboration on the data by all authorized persons at the same time.

Data analysis

We wrote a set of *Jupyter* [14] notebooks with *Python* code to work with the images and wrangle the acquired data. The notebooks were written at the start of the project, to be able to process new scans as soon as they were reconstructed. Re-runs of the notebook added newly scanned and reconstructed data to the analysis, facilitating an efficient quality check of the scans and batched processing of the data.

All analysis notebooks for this work are available online [15].

Preparation for analysis

The main *Jupyter* notebook for this manuscript dealt with reading all log and image files and preparing images for quality checking and further analysis.

At first, *all* log files of *all* the data present in the processed folder were read into a *pandas* [16,17] dataframe. This already enabled us to extract the specimen name and scan, since we performed multiple scans for each specimen, i.e. a low resolution scan with large field of view for the whole head and one or two scans in high resolution focusing on the region of the oral and pharyngeal jaws. From the log files we extracted the relevant values for double-checking the necessary parameters of each scan. All relevant values for each scan were also saved into the dataframe and saved out to the aforementioned table in the Supplementary Materials at the end of each run of the notebook.

After performing 'sanity checks' of the data and reconstruction parameters, we used *Dask* [18] to efficiently access the tomographic data on disk (in the end amounting to a total of nearly a million single images). On average, each of the tomographic datasets contains around 2700 slices, so the total size of the acquired data exceeds the RAM size available on an average high-end workstation.

At first, we extracted the central view of each of the three axial directions of the datasets (i.e. 'anteroposterior', 'lateral' and 'dorsoventral' view) and either saved those to disk or loaded them from disk if they were already generated in prior runs of the notebook. The notebook then also generated the maximum intensity projection (MIP) for each of the anatomical planes and either saved them to disk or loaded them from prior runs.

At the end of the notebook we performed a final sanity check on the MIP images. In this check we examined the mapping of the gray values of the raw projection images to gray values in the reconstructions, i.e. checked that no overexposed pixels are present in the MIP images. This is an efficient way for double-checking the gray value mapping, since the MIP images have already been generated in prior steps of the notebook and contain the highest gray values present in all the reconstructed images of each scan.

Image processing

Extraction of oral and pharyngeal jaws, visualization of tomographic data

To extract the oral jaw (OJ) and pharyngeal jaw (PJ) of the fish, we used *3D Slicer* (Version 4.11.20210226) [19] extended with the *SlicerMorph* tools [20] which aim to help biologists work with 3D specimen data. The reconstructed image stacks were loaded into *ImageStacks*, depending on their size we reduced the image resolution (i.e. downscaled the images) for this first step. The three-dimensional volume was rendered via *VTK GPU Ray Casting*. A custom-made volume property was used as an input to view the scans. Using toggles in the volume rendering, we defined regions of interest (ROIs) for both the OJs and PJs in each specimen. These ROIs were then extracted in their native resolution from the original dataset for further processing. Using the gray value thresholding function in *3D Slicer*'s *Segment Editor*, the teeth in both the oral and pharyngeal jaws were extracted. We used the *Scissor* and *Island* tools of the *Segment Editor* to isolate single regions.

Processed regions of interest were exported as `Nrrd` [21, =Nrrd&oldid=1085264080] files. The three-dimensional visualizations of all regions of interest for each specimen were compiled into overview images (see Figure 1 for an example from the compilation document). In total we compiled overview of 125 specimens with full head morphology, oral jaw and lower pharyngeal jaw profiles.

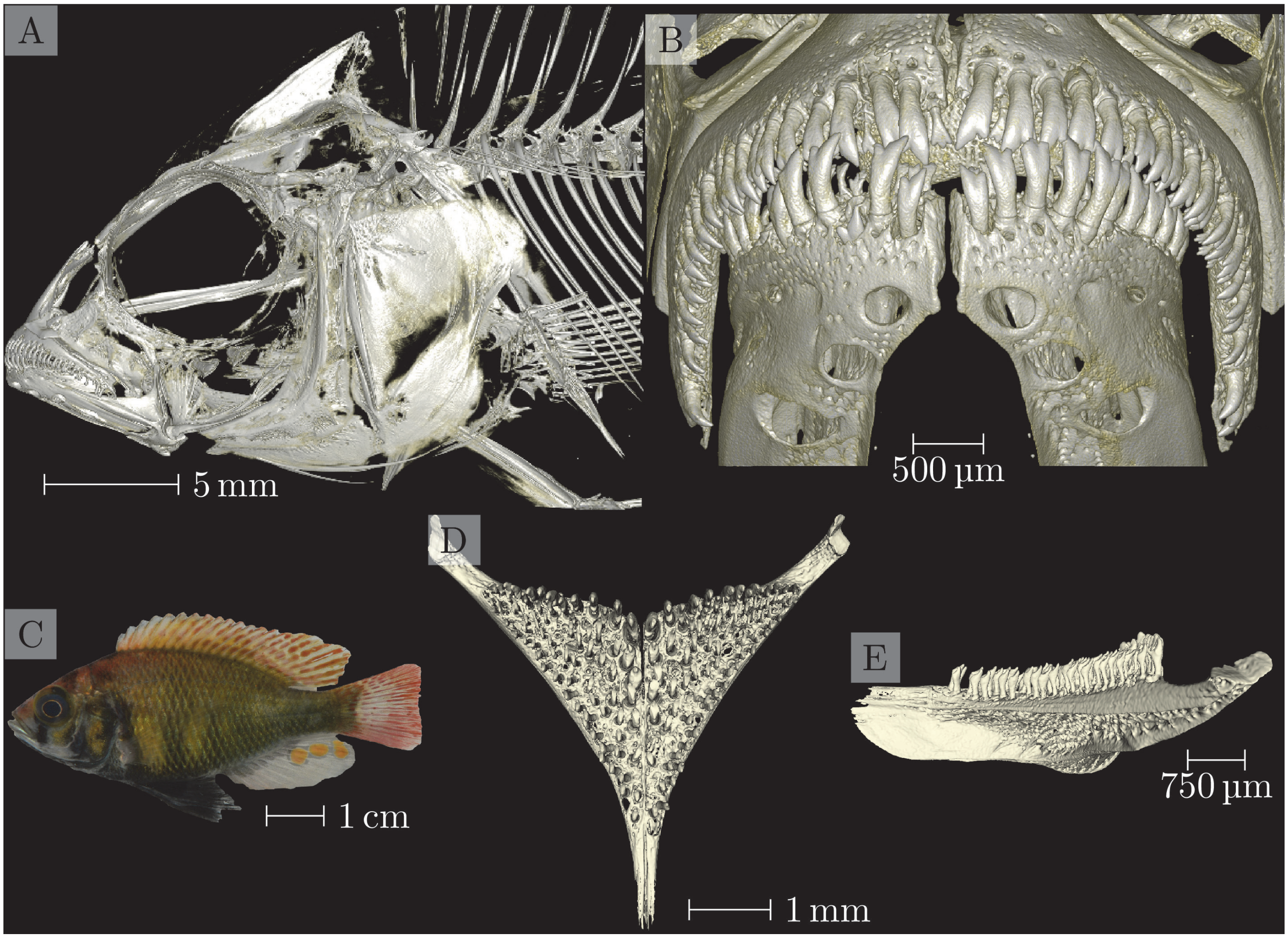

Figure 1: Overview of data from sample 104016, *Enterochromis I cinctus* (St. E). Panel A: Three-dimensional visualization of the head scan. Panel B: Three-dimensional visualization of the oral jaw scan. Panel C: Photograph of the specimen. Panels D and E: Three-dimensional visualization of the pharyngeal jaw, dorsoventral and lateral view, respectively.

Principal components analysis of skull landmarks

Current studies are using 3D geometric morphometrics to compare the morphological shape of these scanned cichlids using statistical analysis. We used a homologous landmark scheme across one-half of the skull for higher density of shape information [7,22], and landmarks were placed on each specimen using *3D Slicer*. To examine differences in shape across the species sampled, we performed a Generalized Procrustes Superimposition on the landmark data to remove the effects of location, size, and rotation from the analysis using the *geomorph* package in *R* (Version 4.2.1) with *RStudio* (Version 2022.07.2+576) [23–25,26,27]. This process brings all specimens to a common origin, scales the landmarks to a unit centroid size, and rotates specimens to reduce distances between landmarks. A principal components analysis was then performed in *geomorph* on the superimposed landmark data to visualize the major axes of shape change across sampled species. We then used phylogenetic information to identify instances of repeated evolution of trophic adaptations in these cichlids.

Automatic extraction of otoliths

Otoliths are structures made up of mostly calcium carbonate located in the head of fishes. Due to their composition, they are easily distinguished in the X-ray images we acquired. We devised an image processing method to automatically and robustly detect the location of the otoliths in the heads of the cichlids we scanned and to extract them from the original data. The whole method is implemented in its own *Jupyter* notebook (part of the aforementioned analysis repository [15]).

Since we took great care to scan the fish parallel to their anteroposterior direction and reconstructed the tomographic datasets parallel to the lateral and dorsoventral direction of the fish we could use this ‘preparation’ for automatically extracting the otoliths the tomographic datasets of the whole fish heads. By extracting both the peaks and the peak widths of the gray values along both the horizontal and vertical direction of the MIP (generated above) we robustly detected the position of the otoliths in the datasets. The robust detection is supported by suppressing a small, configurable part of each region, i.e. the front and back, top and bottom or the flanks.

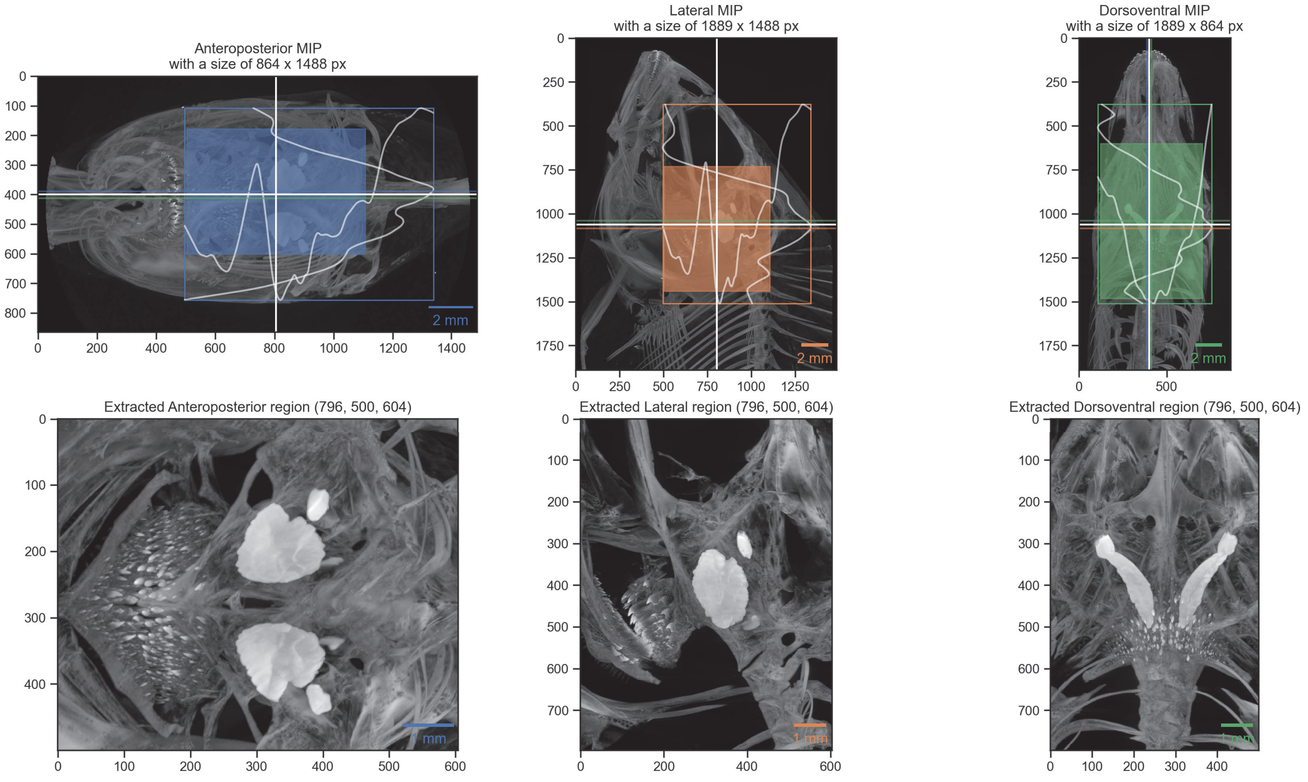

Figure 2: Visualization of automatic otolith extraction. The top row shows the found location of the otolith center in each of the three anatomical directions. The bottom row shows a MIP of each of the three anatomical directions of the extracted otolith region.

Figure 2 shows the visualization of the process. The colored horizontal and vertical bars in each of the directional MIPs denote the found peak location of the two appropriate values. The white bars show the mean of the to detected positions, which was used for extracting the otoliths from the original datasets. Making use of the *Dask* library facilitated efficient access to all the data on disk and writing out small, cropped copies of the datasets around the otolith positions.

By detecting the largest components in the cropped copies of the datasets we can easily extract and visualize the otoliths in 3D, as shown in Figure 3. The extracted otoliths are thus prepared for further analysis and visualization. The simple three-dimensional visualization is integrated in the aforementioned *Jupyter* notebook through an integrated visualization library [28] and is also shown in the Supplementary Materials.

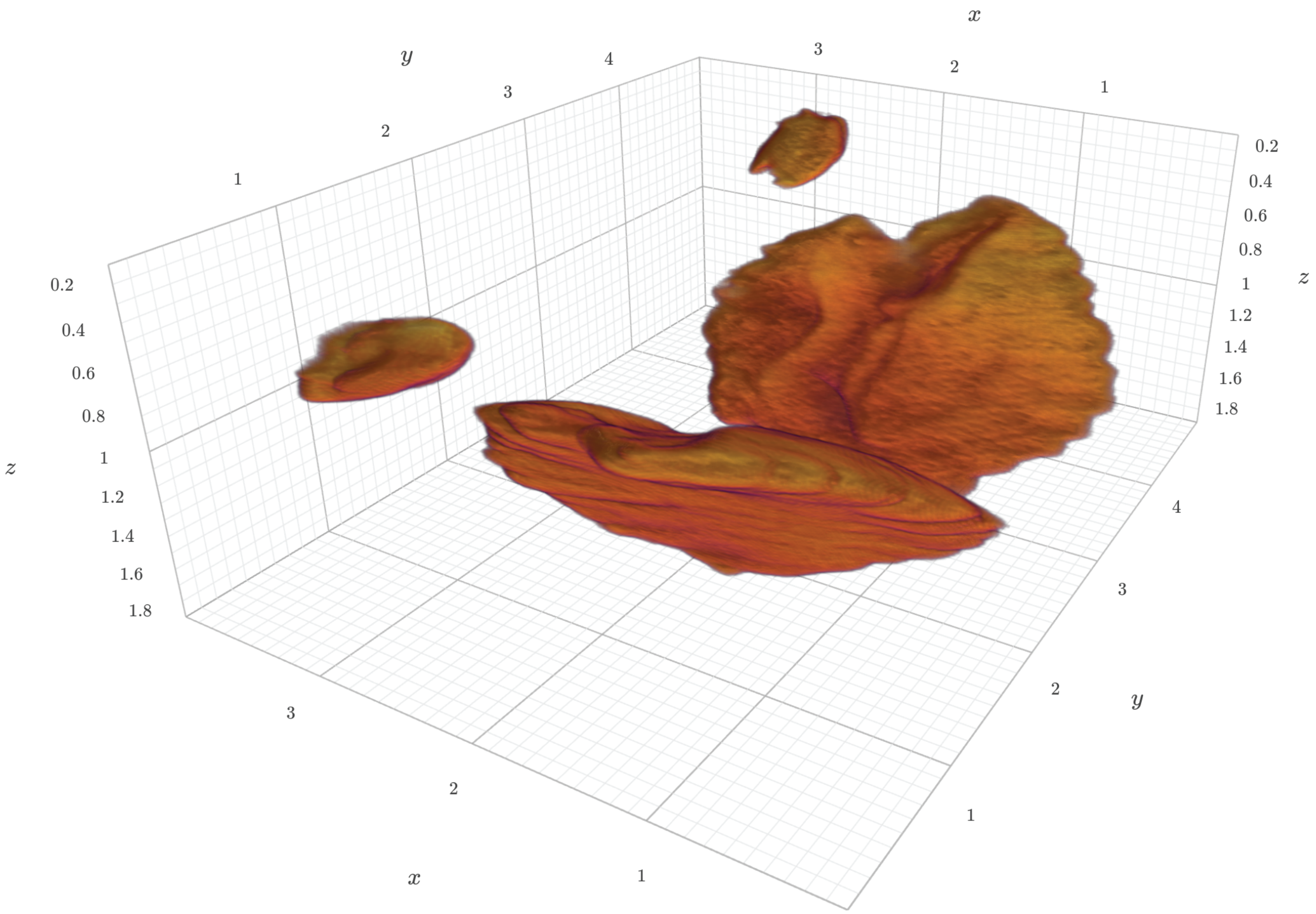

Figure 3: Static three-dimensional view of extracted otoliths of specimen 104016. The specimen was scanned with an isotropic voxel size of 13 µm. The extracted otolith has a size of approximately 250 x 350 x 150 pixels. The axes are labeled in mm steps. A dynamic view of the visualization is available in the Supplementary Materials.

The notebook for extracting the otoliths can be run in your browser without installing any additional software (via *Binder* [29]). To do this, one starts the notebook by clicking a single button in the README file of the project repository [15]. This starts a computing environment in the cloud, downloads the tomographic data we acquired of *one* specimen, and performs both the otolith extraction and visualization in your browser.

Discussion

We acquired high resolution tomographic datasets of a large collection of cichlids. The acquired datasets were imaged over a wide-spanning range of voxel size (3.5–50 μm) permitting both the analysis of finest details we wanted to resolve (i.e. the structure of the teeth) and having datasets spanning the whole region of interest of the fish (i.e. the whole head for principle components analysis).

Imaging and preparation for analysis

The whole study we presented here spanned a *long* time frame. It was thus paramount to run the imaging process and the preparation of the tomographic datasets in a batched mode. The *Jupyter* notebook written to prepare the datasets for quality control and analysis were facilitating a short turnaround time for feedback on single scans and such a batched analysis.

Otolith extraction

The method to extract the otoliths from the tomographic dataset works robustly for all of the different fish sizes and shapes. The extraction is robust because it is based on a combination of distinct details of the gray value curve over the different anatomical directions. The details of the otolith extraction method have been extensively tuned and run in a fully automatic way. This allows a highly reproducible and unbiased extraction of the otoliths from the tomographic datasets. This is even the case for one fish which was scanned with still a hook in his mouth, where the otoliths were nonetheless extracted automatically.

Data on such automatically extracted otoliths, like volume and geometric information like eccentricity and moments of inertia is biologically interesting as the otoliths grow with the age of the fish. One could help estimate the age of wild fishes using a calibration based on the otolith measurements of a fish of known age. It is worth nothing that estimation of age of tropical fishes is not as simple as for fishes from temperate regions where one can distinguish summer and winter layers within the otolith.

Outlook

The acquired tomographic datasets are the basis for multiple additional analyses of fish morphology.

The presented method offers an insight and algorithm on how to perform tomographic scans, preview and analyze micro-computer tomographic datasets of a large collection of fish. The workflow is relying only on free and open-source software and can thus be used and verified independently by any interested reader. All the *Jupyter* notebook described herein is also freely available online [15].

Author Contributions

Contributor Roles Taxonomy, as defined by the National Information Standards Organization.

Author	Contributions
David Haberthür	Conceptualization, Data curation, Formal Analysis, Investigation, Methodology, Project administration, Software, Visualization, Writing – original draft, Writing – review & editing
Mikki Law	Data curation, Investigation, Methodology, Project administration, Visualization, Writing – review & editing
Kassandra Ford	Investigation, Methodology, Visualization, Writing – review & editing
Marcel Häsler	Investigation, Project administration, Resources, Writing – review & editing
Ole Seehausen	Conceptualization, Investigation, Resources, Supervision, Writing – review & editing
Ruslan Hlushchuk	Conceptualization, Resources, Supervision, Writing – review & editing

Acknowledgments

We thank Salome Mwaiko for taking care of the fish collection at Eawag and Mark Charran for helping to find suitable specimens to represent each species. We are grateful to the Microscopy Imaging Center of the University of Bern for the infrastructural support. We thank the `manubot` project [30] for helping us write this manuscript collaboratively.

Supplementary Materials

Parameters of tomographic scans of all the fishes

The CSV file ScanningDetails.csv gives a tabular overview of all the (relevant) parameters of all the scans we performed. This file was generated with the data processing notebook and contains the data which is read from *all* the log files of *all* the scans we performed. A copy of each log file is available in a folder in the data processing repository.

Three-dimensional view of *one* of the extracted otoliths

The three-dimensional view of sample 104016 was generated in the otolith extraction notebook and saved as a self-contained `HTML` file with *K3D-jupyter*. A copy of this `HTML` file can be viewed and interacted with through the GitHub HTML preview.

References

1. Kocher TD. Adaptive evolution and explosive speciation: the cichlid fish model. Nat Rev Genet. 2004;5: 288–298. doi:10.1038/nrg1316

2. Seehausen O. African cichlid fish: a model system in adaptive radiation research. Proc R Soc B. 2006;273: 1987–1998. doi:10.1098/rspb.2006.3539

3. Liem KF. Evolutionary Strategies and Morphological Innovations: Cichlid Pharyngeal Jaws. Systematic Zoology. 1973;22: 425. doi:10.2307/2412950

4. Seehausen O. Process and pattern in cichlid radiations – inferences for understanding unusually high rates of evolutionary diversification. New Phytol. 2015;207: 304–312. doi:10.1111/nph.13450

5. Ford KL, Albert JS, Summers AP, Hedrick BP, Schachner ER, Jones AS, et al. A New Era of Morphological Investigations: Reviewing Methods for Comparative Anatomical Studies. Integrative Organismal Biology. 2023;5. doi:10.1093/iob/obad008

6. Messerli M, Aaldijk D, Haberthür D, Röss H, García-Poyatos C, Sande-Melón M, et al. Adaptation mechanism of the adult zebrafish respiratory organ to endurance training. Shelden EA, editor. PLoS ONE. 2020;15: e0228333. doi:10.1371/journal.pone.0228333

7. Ford KL, Peterson R, Bernt M, Albert JS. Convergence is Only Skin Deep: Craniofacial Evolution in Electric Fishes from South America and Africa (Apteronotidae and Mormyridae). Integrative Organismal Biology. 2022;4. doi:10.1093/iob/obac022

8. Available: https://osf.io/ecmz4

9. OpenSCAD models. TomoGraphics; 2022. Available: https://github.com/TomoGraphics/Hol3Drs/blob/dedca665a77fa782c103eed63b866b2cceb30de9/STL/EAWAG.Fish.stl

10. Haberthür D. TomoGraphics/Hol3Drs: A release. Zenodo; 2019. doi:10.5281/zenodo.2587555

11. OpenSCAD. [cited 24 Apr 2023]. Available: https://openscad.org

12. OpenSCAD models. TomoGraphics; 2022. Available: https://github.com/TomoGraphics/Hol3Drs/blob/dedca665a77fa782c103eed63b866b2cceb30de9/EAWAG.Fish.scad

13. Haberthür D. Jupyter notebooks for image processing and data analysis of EAWAG Cichlids project. 2023. Available: https://github.com/habi/EAWAG/blob/e5d6be6b416b7c66eaf72a563b0ce5648569f54d/rsync-fishes.sh

14. Kluyver T, Ragan-Kelley B, Pérez F, Granger B, Bussonnier M, Frederic J, et al. Jupyter Notebooks – a publishing format for reproducible computational workflows. In: Loizides F, Scmidt B, editors. IOS Press; 2016. pp. 87–90. doi:10.3233/978-1-61499-649-1-87

15. Haberthür D. habi/EAWAG: Jupyter notebooks for preparing tomographic scans of a collection of cichlids for analysis. Zenodo; 2022. doi:10.5281/zenodo.6798632

16. Team TPD. pandas-dev/pandas: Pandas. Zenodo; 2022. doi:10.5281/zenodo.7093122

17. McKinney W. Data Structures for Statistical Computing in Python. Proceedings of the Python in Science Conference. SciPy; 2010. doi:10.25080/majora-92bf1922-00a

18. Dask Development Team. Dask: Library for dynamic task scheduling. 2016. Available: https://dask.org

19. Fedorov A, Beichel R, Kalpathy-Cramer J, Finet J, Fillion-Robin J-C, Pujol S, et al. 3D Slicer as an image computing platform for the Quantitative Imaging Network. Magnetic Resonance Imaging. 2012;30: 1323–1341. doi:10.1016/j.mri.2012.05.001

20. Rolfe S, Pieper S, Porto A, Diamond K, Winchester J, Shan S, et al. SlicerMorph: An open and extensible platform to retrieve, visualize and analyse 3D morphology. Methods Ecol Evol. 2021;12: 1816–1825. doi:10.1111/2041-210x.13669

21. Main Page. Wikipedia, the free encyclopedia. 2022. Available: https://en.wikipedia.org/w/index.php?title=Main_Page&oldid=1114291180

22. Ford KL, Bernt MJ, Summers AP, Albert JS. Mosaic Evolution of Craniofacial Morphologies in Ghost Electric Fishes (Gymnotiformes: Apteronotidae). Ichthyology & Herpetology. 2022;110. doi:10.1643/i2021016

23. Collyer ML, Adams DC. RRPP: An <scp>r</scp> package for fitting linear models to high-dimensional data using residual randomization. Freckleton R, editor. Methods Ecol Evol. 2018;9: 1772–1779. doi:10.1111/2041-210x.13029

24. Adams DC, Collyer ML, Kaliontzopoulou A, Baken EK. Geomorph: Software for geometric morphometric analyses. R package version 4.0.4. 2022. Available: https://cran.r-project.org/package=geomorph

25. Collyer ML, Adams DC. RRPP: Linear model evaluation with randomized residuals in a permutation procedure, r package version 0.6.2. 2021. Available: https://cran.r-project.org/package=RRPP

26. R Core Team. R: A language and environment for statistical computing. Vienna, Austria: R Foundation for Statistical Computing; 2018. Available: https://www.R-project.org/

27. RStudio Team. RStudio: Integrated development environment for r. Boston, MA: RStudio, PBC.; 2022. Available: https://www.rstudio.com/

28. K3D Jupyter. K3D-tools; 2023. Available: https://github.com/K3D-tools/K3D-jupyter

29. Jupyter P, Bussonnier M, Forde J, Freeman J, Granger B, Head T, et al. Binder 2.0 - Reproducible, interactive, sharable environments for science at scale. Proceedings of the Python in Science Conference. SciPy; 2018. doi:10.25080/majora-4af1f417-011

30. Himmelstein DS, Rubinetti V, Slochower DR, Hu D, Malladi VS, Greene CS, et al. Open collaborative writing with Manubot. Schneidman-Duhovny D, editor. PLoS Comput Biol. 2019;15: e1007128. doi:10.1371/journal.pcbi.1007128
