# Supplementary material for: Microtomographic investigation of a large corpus of cichlids
Source: PLoS One. 2023 Sep 27;18(9):e0291003. doi: 10.1371/journal.pone.0291003 (PMC10529598; doi:10.1371/journal.pone.0291003)
Supplement: Attachment — Submitted filename: Answer.pdf [file pone.0291003.s004.pdf]

---
title: Answer to reviewers of PONE-D-23-10896 (Microtomographic investigation of a large corpus of cichlids)
author: David Haberthür
date: 17.7.2023

Review Comments to the Author

> Reviewer #1: Excellent paper.
> It was not clear if you could discern daily rings on the otoliths.
> You might want to discuss if you can use rings on otoliths to age the fish without removing them.
> Also, do you have to stain the fish to use these rings?

The data shown in this manuscript is from unstained specimen.
They have been stored in ethanol and imaged in wet foam to avoid any drying during imaging, but *no* staining was applied.
Since we here only focus on the morphology of the skull and teeth such a staining was not necessary to extract the needed data from the tomographic images.
Growth rings would most probably be visible in the analyzed specimens, but only if some staining is applied, e.g. by storing the fish in Lugol solution.
Since this was *not* the focus of the present study we did not do this here.

To use the data on both growth rings and volume of the otoliths one would have to calibrate this first to be able to extract age estimates.
I.e. one would have to make a simple growth experiment where a number of fish with known age are euthanized, tomographically imaged and then the volume of their extracted otoliths is correlated with their age.
Such a calibration would match size and volume of both the (lab-raised) fish and the otoliths with the age of the fish.
This data could then be used to estimate the age of fishes collected in the wild.

> Reviewer #2: I have carefully evaluated the manuscript titled "Microtomographic investigation of a large corpus of cichlids". The authors present a substantial collection of tomographic images from cichlids collected in Lake Victoria, Africa. They showcase significant cranial, maxillary, and otolith images from 362 specimens and provide open-source Python notebooks to facilitate the manipulation and extraction of specific parts. I believe that this work represents a significant contribution to the understanding of cichlids and the application of Micro-CT imaging in fish studies. However, I have some suggestions to improve the manuscript:

Thank you very much for the kind words.
As we (now) state in the [imaging section](https://habi.github.io/EAWAG-manuscript/#micro-computed-tomographic-imaging) we acquired 372 single tomographic scans of 133 different specimen (updated since submission of the manuscript, as a rest of fish relevant for the study was added).
This means that each specimen was scanned several times, usually one lower-resolution scan of the full head and two single higher-resolution scans for the oral and pharyngeal jaws.

> The introduction section needs improvement.
> It is too simplistic and lacks informative details about the state of the art and the justification for the work.

We've deliberately kept the introduction rather terse.
The justification of the work lies in the non-destructive imaging of unstained samples.
We have expanded the introduction a bit and added references to manuscripts detailing microtomographic imaging in the life sciences.
As this manuscript is not a review, we kindly refuse to add more information on the background of microtomographic imaging.

> Merging the Materials and Methods section with the Results is not a reasonable way to present the work.
> It would be better to separate them for clarity.

The manuscript is focused on the method on how to acquire and assess tomographic datasets of a large corpus of cichlids.
Especially for the image processing part, the method and results are highly intertwined as the Python code directly delivers the results.
We believe that the approach we chose here here is the most appropriate way to present the whole data data acquisition and analysis pipeline and kindly ask the reviewer to accept our choice.

> The section on Micro-computed tomographic imaging should be the core of the paper and explained in more detail. The authors have extensive experience scanning 362 fish specimens of different species and sizes. Therefore, a detailed imaging protocol that can assist readers in obtaining new images should be provided. For example, what criteria did the authors use to select the best set of parameters? It is clear and expected that larger fish would have lower resolution (larger voxel size), but how does the relationship between size and parameters work? How was the filter thickness chosen?

As stated above and in the text, we acquired 372 single tomographic scans of 133 different specimen.

> The first sentence of the last paragraph of the subsection Micro-computed tomographic imaging is unclear: "While performing the work, a subset of the data was always present on the production system, for working with it (see Preparation for analysis below)".
> Please provide more precise information.

We are sorry for the imprecise description of the data archival and copying process.
The last paragraph of [this section](https://habi.github.io/EAWAG-manuscript/#micro-computed-tomographic-imaging) was expanded and improved.

> The authors should revise the entire "Data analysis" subsection to present a more didactic version that helps any reader use the notebooks.
> Currently, it seems that only Python users with some expertise can understand what needs to be done.
> Perhaps the authors could provide a guide or tutorial in the GitHub repository.

The notebooks are written in Python, and are presented as Jupyter notebooks.
This makes them accessible 'piece wise', e.g. each part of the code is didactically separate and somehow compartmentalized.
The code is written in Python, so some experience with it is beneficial to thoroughly understand it.
The code and notebooks are also prepared in a way that *no* installation of *any* software except an up-to date web browser is necessary to test it.
This testing can be done by clicking one button in the code repository (linking to https://mybinder.org/v2/gh/habi/eawag/HEAD) to launch a Jupyter environment in the cloud, which is also already explained in the last paragraph before the [Discussion section](https://habi.github.io/EAWAG-manuscript/#discussion).
We understand the concerns of the reviewer though, and added explanation text and comments to the notebooks guiding (first time) users of the notebooks.
All these updates happen in [the code repository](https://github.com/habi/EAWAG) and have *not* been specifically mentioned in the manuscript text.
Two of the notebooks ([DataWrangling](https://nbviewer.org/github/habi/EAWAG/blob/main/DataWrangling.ipynb) and [DataSize](https://nbviewer.org/github/habi/EAWAG/blob/main/DataSize.ipynb)) were used to help us generate 'checking' and 'collaboration' files (DataWrangling) or used to help us generate text for the manuscript, i.e. to extract exact details on sample numbers, total amount of projections and reconstructions and their exact size on disk (DataSize).

As these notebooks are not explicitly mentioned in the manuscript they are not extensively commented (only commented).
The two notebooks mentioned in the text ([DisplayFishes](https://nbviewer.org/github/habi/EAWAG/blob/main/DisplayFishes.ipynb)) and ([ExtractOtoliths](https://nbviewer.org/github/habi/EAWAG/blob/main/ExtractOtoliths.ipynb)) have been updated with more comments and explanatory text between the logical steps.
These additions are [visualized in the respective GitHub repository](https://github.com/habi/EAWAG/compare/v1.1...HEAD), but not specifically mentioned in the text.
We slightly expanded the description of the notebooks at the start of the [Data analysis section](https://habi.github.io/EAWAG-manuscript/#data-analysis) though.

> How was the sanity check performed (as mentioned in the second paragraph of the subsection "Preparation for Analysis")?

The mentioned 'sanity check' was performed manually, e.g. by looking at all the values (mostly the reconstruction parameters), to exclude any operator error.
If errors were spotted at this stage, the reconstructions were deleted and re-done with the correct parameters.
Sometimes errors or problems were only spotted at a later stage, when either the pharyngeal or oral jaws were extracted, or when we noticed that some part of the the whole skull were out of the field of view of the tomographic scan prior to performing the PCA analysis mentioned later in the manuscript.
This means that specifying this as 'sanity check' is more of a glorified wording of telling the readers that we manually looked at the values of each performed tomographic scan to exclude and catch operator errors.
We reworded the sentence to hopefully make this more clear.

> In the same paragraph, the authors mentioned that each tomographic dataset contains around 2700 slices, exceeding the available RAM size on an average high-end workstation.
> Could you clarify if this limitation affected the analysis and its implications?

We wrote that *the total size of the acquired data exceeds the RAM* of any workstation (e.g. around 1.5 TB).
One or more single datasets would fit into RAM very well, It is not possible to load *all* data concurrently for analysis.
The use of *Dask* and more specifically [*dask-image*](@https://image.dask.org/) for loading only the currently used data for each specimen from the HD (or remote storage) into RAM on demand made working with the *all* the data feasible.
We have updated the sentence to explain this.

> The Wikipedia citation in the second paragraph of the subsection "Extraction of oral and pharyngeal jaws..." is not clear.
>Please provide more specific information or replace the citation with a more appropriate reference.

The Wikipedia citation to a [date-specific version of the `Nrrd` article on Wikipedia](https://w.wiki/5mBK) was not correctly expanded by our manuscript preparation system.
We replaced the Wikipedia link with a more appropriate link to [the `Nrrd` format definition](https://teem.sourceforge.net/nrrd/format.html) and thank the reviewer for noticing this issue.

> Regarding the sentence "In total, we compiled an overview of 125 specimens with full head morphology, oral jaw, and lower pharyngeal jaw profiles," why was this done only for a subset of the sample? Please explain the rationale.

As stated above and in the text, we actually acquired 372 single tomographic scans of 133 different specimen.
It is correct though that "125" here is wrong, since we've acquired more scans after submission of the manuscript.

This has been corrected, and we thank the reviewer for noticing this.

> The results of the subsection "Principal components analysis of skull landmarks" should be presented in a more accessible manner.
> Consider providing a tutorial or guide for readers to better understand and apply the analysis.
> As it currently stands, this subsection may be considered irrelevant for the paper.

This subsection is currently being 'expanded' into a self-contained publication.
We deliberately only very briefly mentioned the method here, as the upcoming publication is to be submitted soon.

> The procedure for detecting and extracting otoliths in the subsection "Automatic extraction of otoliths" is not clear.
> Please provide clearer instructions or guidelines for readers using the notebook.

As mentioned above, the Jupyter notebook used to detect the otolith position and to extract the otoliths from the scans covering the whole skull are extensively commented.
We have added more explanatory comments and text to the notebook on GitHub, increasing their 'didactiveness'.

> Use the full words, rather than abbreviations, in figure legends for clarity.

Abbreviations in the legends have been written-out (except SI units).
Additionally we added a bit of background information on the specimen to the legend of Fig. 1.

> In the discussion section, the authors mentioned acquiring high-resolution tomographic datasets of a large collection of cichlids.
> However, the statement that "the acquired datasets were imaged over a wide-spanning range of voxel size (3.5–50 μm)" is incorrect.
> The voxel size is specimen-specific, depending on the size of the fish and the chosen parameters.
> Please clarify this point to accurately represent the imaging process.
> The finest resolution was obtained only for small fishes, so specific structures of a fish will have the same image resolution throughout the entire specimen.

The statement over the 'wide-spanning range of voxel sizes' is *not* incorrect, at most---if applied to *one* specimen---imprecise.
The range of voxel sizes is true for the whole study, while---as also correctly noted by the reviewer---the voxel size is specimen- or scan-specific.
We have added a bit of explanatory text to both the materials and discussion section.

> It would be beneficial to include references in the discussion section to support the arguments and provide a broader context for the findings.

The main scientific 'use case' of the method focuses on the automated, non-destructive extraction of the otolith volume from the tomographic data.
To the best of our knowledge, our manuscript is the first to describe such a method.
We have found two references describing otoliths or otolith structure, both on explanted otoliths.
We have added those to the discussion section.

> Overall, the study seems to have spanned a considerable time frame.
> However, the manuscript's content and structure need improvement to reach a wider audience and effectively communicate the significance of the work.
> The authors have provided a valuable set of open-source Python notebooks, which is commendable and will be highly useful for the scientific community.

> However, the workflow needs to be better explained to enable readers to follow the procedures accurately.

As mentioned above, we have improved both the text and the Jupyter notebooks (linked in the text) with explanatory sentences and comments.

> By addressing these suggestions, the manuscript will become more comprehensive, accessible, and representative of the valuable contribution made by the authors.

We thank the reviewer for the kind words and the time spent on improving our work with relevant questions and comments.
The manuscript has considerably improved by the updates we have performed.
